# Knowledge-based quality assurance of a comprehensive set of organ at risk contours for head and neck radiotherapy

**DOI:** 10.3389/fonc.2024.1295251

**Published:** 2024-02-29

**Authors:** Jamison Brooks, Erik Tryggestad, Aman Anand, Chris Beltran, Robert Foote, J. John Lucido, Nadia N. Laack, David Routman, Samir H. Patel, Srinivas Seetamsetty, Douglas Moseley

**Affiliations:** ^1^ Department of Radiation Oncology, Mayo Clinic Rochester, Rochester, MN, United States; ^2^ Department of Radiation Oncology, Mayo Clinic Arizona, Phoenix, AZ, United States; ^3^ Department of Radiation Oncology, Mayo Clinic Florida, Jacksonville, FL, United States

**Keywords:** contour review, quality assurance, automation, radiotherapy, outlier detection

## Abstract

**Introduction:**

Manual review of organ at risk (OAR) contours is crucial for creating safe radiotherapy plans but can be time-consuming and error prone. Statistical and deep learning models show the potential to automatically detect improper contours by identifying outliers using large sets of acceptable data (knowledge-based outlier detection) and may be able to assist human reviewers during review of OAR contours.

**Methods:**

This study developed an automated knowledge-based outlier detection method and assessed its ability to detect erroneous contours for all common head and neck (HN) OAR types used clinically at our institution. We utilized 490 accurate CT-based HN structure sets from unique patients, each with forty-two HN OAR contours when anatomically present. The structure sets were distributed as 80% for training, 10% for validation, and 10% for testing. In addition, 190 and 37 simulated contours containing errors were added to the validation and test sets, respectively. Single-contour features, including location, shape, orientation, volume, and CT number, were used to train three single-contour feature models (z-score, Mahalanobis distance [MD], and autoencoder [AE]). Additionally, a novel contour-to-contour relationship (CCR) model was trained using the minimum distance and volumetric overlap between pairs of OAR contours to quantify overlap and separation. Inferences from single-contour feature models were combined with the CCR model inferences and inferences evaluating the number of disconnected parts in a single contour and then compared.

**Results:**

In the test dataset, before combination with the CCR model, the area under the curve values were 0.922/0.939/0.939 for the z-score, MD, and AE models respectively for all contours. After combination with CCR model inferences, the z-score, MD, and AE had sensitivities of 0.838/0.892/0.865, specificities of 0.922/0.907/0.887, and balanced accuracies (BA) of 0.880/0.900/0.876 respectively. In the validation dataset, with similar overall performance and no signs of overfitting, model performance for individual OAR types was assessed. The combined AE model demonstrated minimum, median, and maximum BAs of 0.729, 0.908, and 0.980 across OAR types.

**Discussion:**

Our novel knowledge-based method combines models utilizing single-contour and CCR features to effectively detect erroneous OAR contours across a comprehensive set of 42 clinically used OAR types for HN radiotherapy.

## 1 Introduction

Standardized and precise organ at risk (OAR) contours are essential for head and neck (HN) radiation therapy, enabling safe treatments and more consistent dose reporting (1). While manual contouring is time-consuming and prone to user variation, Deep learning (DL) autocontouring methods have demonstrated time savings (2, 3) and reduced variation (4, 5) compared to manual contouring methods. Autocontouring tools generally perform well, however, a variety of clinically relevant failures, ranging from minor to severe, do occur with no warning given from the model-hosting tool (**Supplementary Figure S1**). Consequently, both contours created manually and with autocontouring tools require thorough quality assurance (QA) review by trained personnel to ensure safe and effective radiotherapy treatments.

The ability of DL autocontouring tools to quickly create many contours enables more contours to be used for a given treatment site and expedites both offline and online adaptive treatment planning. However, it also increases the amount of time spent reviewing contours. Automated approaches to contour review may be able to both decrease review time and improve consistency (6, 7), making them a desirable potential tool for clinical use. Such approaches could be deployed on their own, or in combination with human reviewers to assist them in identifying contours of poor quality.

Several automated algorithmic methods have been proposed for automated OAR contour QA (8–11). One of the most popular approaches utilizes a set of features calculated from high-quality contours to classify contours of unknown quality as similar (acceptable) or different (erroneous). This is referred to as knowledge-based outlier detection using one-class training. Features for this approach include contour volume, shape, orientation, position, and image characteristics. Models for knowledge-based contour classification include statistical approaches looking at several features independent of one another (univariate statistical models) (12, 13), as well as multivariate statistical models, and DL models (14, 15). Most knowledge-based outlier detection methods for OAR contour QA have relied on a few hand-selected features for evaluation which are largely informed by domain experts in radiation oncology. This expertise may allow for comparable performance between simpler statistical models and DL models. Despite several publications, it remains unclear how the performance of univariate models, multivariate statistical models, and DL models compares for knowledge-based OAR contour QA.

In previous studies, knowledge-based contour outlier detection models have used features describing the relationships between different OAR types (henceforth referred to as contour-to-contour relationships or CCRs) to minimize patient-to-patient variation and detect erroneous contours (14–16). Ensuring that contours are appropriately separate, touching, or overlapping is crucial for HN radiation treatment planning due to the precise relationships between many OARs. Neglecting to do so can lead to inaccurately contoured anatomy and unreported dose to OARs because of contour gaps between anatomically touching OARs during IMRT optimization. While CCR relationships are both quantifiable and important, we are not aware of any studies that have directly evaluated the effectiveness of features that quantify contour separation and overlap for the detection of erroneous contours.

To ensure the usefulness of an automated OAR contour quality assurance tool for a specific treatment site, it ideally should have acceptable performance that generalizes to many OAR types (brain, left lung, larynx, etc.) and should encompass various disease types, and patient anatomies. For HN treatment sites, as many as 42 OAR types have been reported to be relevant for HN treatment planning (3). However, existing knowledge-based contour QA studies that have evaluated individual OAR types, assess no more than 17 in any given study (9, 10, 12, 14, 16). This limitation may be attributed to the lack of standardized and curated contours available for model training. Analysis of additional OAR types for HN is needed to demonstrate whether knowledge-based contour outlier detection models can be used for any clinically relevant HN OAR types.

This study investigates the performance and generalizability of knowledge-based, outlier detection methods to identify erroneous contours for 42 HN OAR types used clinically for radiotherapy. This is the largest number of OAR types evaluated for HN in a single study to date. Model training was performed using manually contoured, highly curated, contour sets derived from patients with HN cancer being treated with radiotherapy. Three single-contour feature model types that have not been compared for contour outlier detection in previous work, a univariate statistical model (z-score) (12), a multivariate statistical Mahalanobis distance (MD) (17, 18) model, and a DL autoencoder (AE) model (19), are compared to identify the model type with the best performance and generalizability to each HN OAR type. As a secondary aim, the study investigates the potential of a novel CCR model, that assesses contour separation and overlap, in combination with the three compared models to enhance performance.

## 2 Materials and methods

### 2.1 Data curation and allocation

The study utilized retrospectively collected data from patients with HN cancers who underwent radiotherapy at Mayo Clinic Rochester and Mayo Clinic Arizona between 2016 and 2020. The dataset encompassed a diverse range of HN disease sites and progressions, including patients with prior resection, representing the current treatment landscape at the institutions. CT images were acquired at simulation before the start of radiotherapy treatment using multiple Somatom Definition AS (Siemens, Munich Germany) CT scanners with voxel dimensions of 1.27 mm x 1.27 mm x 2 mm. The CT images were acquired at 120 kVp and most were reconstructed using iterative metal artifact reconstruction techniques to minimize artifacts caused by dental fillings or other metallic objects commonly present during HN radiotherapy. All CT scanners underwent monthly testing using a CatPhan® phantom *(Phantom Laboratory, Salem New York)* to ensure Hounsfield Unit accuracy (**Supplementary Methods** and **Supplementary Table S1**). Head and neck planning CT images and contours used for patient treatment were retrospectively selected and curated to ensure they adhered to institutional guidelines for standardization. This included physician, dosimetrist and physics review and editing during retrospective curation. A thorough description of the dataset and curation efforts has been published (20). The dataset, comprising 490 patient structure sets with corresponding CT images, was considered the gold-standard acceptable patient dataset. These sets were divided into training (80%), validation (10%), and test (10%) subsets. The use of retrospective HN patient data for model training was deemed exempt by our institutional IRB.

Before assessing the performance and generalizability of knowledge-based outlier detection methods to detect erroneous contours, it is essential that the erroneous contours evaluated reflect errors that commonly result from clinical failures. Such errors can occur from both manually created contours, or contours generated using autosegmentation tools. We identified four main categories of such failures that occur: boundary errors, volume errors, non-adjacent slice errors, and positional errors.

Boundary errors encompass instances of accidental border expansion or subtraction, poor delineation of anatomical boundaries, incorrect identification of boundaries based on HU-intensity thresholding, or incorrect propagation of contours from one image set to another due to small deformable or rigid image registration errors.

Volume errors encompass the addition or removal of volumes from an OARs correct volumes. Unlike boundary errors, which pertain to inaccuracies in contour borders, volume errors result from the addition or subtraction of convex shapes from the correct contour. These errors can occur due to incorrect definitions of anatomical boundaries, incomplete contouring, disconnected volumes, or the improper identification of the slices where a contour should start and end.

Non-adjacent slice errors occur due to inadvertent selections of single-slice volumes (i.e. ‘misclicks’) or inconsistent and ‘jagged’ delineations of contour boundaries from one slice to another, which may occur during contouring.

Positional errors represent errors resulting in the central location of a contour being substantially misplaced. Such errors arise from mismatched structure labels, errors in manual identification of OARs, or errors made by CT autocontouring tools. Such errors made by autocontouring tools have been observed during the analysis of CT images with abnormal anatomy, positioning, or CT values for several FDA-approved autocontouring tools evaluated by the authors.

### 2.2 Data augmentation

After identifying these common clinical failure modes, manually generated erroneous contours were introduced *ad hoc* by a medical physicist (JB) who edited gold-standard acceptable contours to mimic clinically observed errors encountered during both manual contouring and autocontouring processes. Manually generated erroneous contours were added directly to the validation and test sets after creation. Each erroneous contour error was additionally categorized as moderate or major by the contour editor, providing the ability to assess how the clinical severity of errors influenced the performance of automated outlier detection. Errors categorized as moderate may or may not be clinically relevant depending on clinical context such as the treatment planning approach and the relationship with the target, while errors categorized as major would be relevant in nearly all clinical contexts.

The total number of contours with boundary, volume, non-adjacent slice, and position errors were 74, 99, 14, and 40 respectively. The total number of contours with major and minor errors were 111 and 116. Error types were distributed randomly across OAR types. In the validation set, a minimum of four erroneous contours were created for each OAR type that had left or right counterparts (i.e. left and right lung), and a minimum of five erroneous contours were created for all other OAR types (i.e. brain, larynx, etc.).

### 2.3 Overview of knowledge-based QA framework

**Figure 1:**
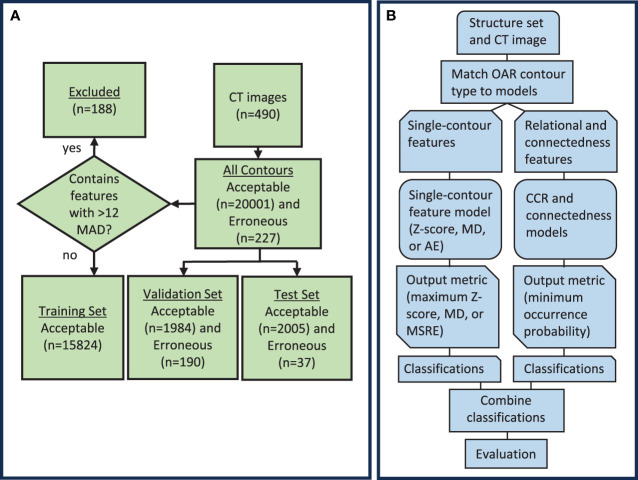
**(A)** Diagram showing the distribution of data used to create and evaluate the knowledge-based QA framework. **(B)** Workflow diagram for the knowledge-based QA framework. AE, Autoencoder; MD, Mahalanobis Distance; CCR, contour-to-contour relationship; MSRE, mean squared reconstruction error.

In this study, the knowledge-based QA framework was developed through several steps (**Figures 1A, B**). First, the training dataset, which consisted of acceptable contours only, was separated by OAR type (i.e. brain, left eye, esophagus). Then, the desired features were calculated for each contour, and model training was performed. Features dependent on only one contour were assigned to each of the three single-contour feature model types (AE, MD, and Z-score), while features involving the relationship between two contours were assigned to the CCR model type. A feature counting the number of disconnected parts in a single contour was included as an additional statistical check outside of the other models as the connectedness model. Separate single-contour feature, CCR, and connectedness models were trained for each OAR type. All models generated a single output metric that indicated the likelihood of a contour being erroneous. Output metrics from models of the same model type were thresholded using a single value to obtain classifications. After model training, the validation set, which included both acceptable and erroneous contours, was used to evaluate the model’s performance, select input features, and determine output metric thresholds. Classifications obtained from the single-contour feature models, CCR models, and connectedness models were combined to form the final classification. Lastly, to ensure no overfitting, the models were evaluated on the test set.

### 2.4 Single-contour feature selection

Feature selection for the single-contour feature models was performed initially by choosing features describing contour shape, volume, location, orientation, and CT number. Features were selected to be generalizable to a wide variety of OAR types and were based on common features used in the literature (12–16). A total of 44 features were included and the Pearson correlation coefficient (21) was used to identify and remove features that were strongly correlated, either positively or negatively, across all OARs (**Supplementary Figure S2**). This was when the correlation was greater than approximately ±0.7. Feature reduction was performed using the validation set to reduce feature correlation while maintaining high classification performance for single-contour feature models. After single-contour feature determination, the same feature set was used for all OAR types and model types (**Table 1**). This was done to identify a set of features that would generalize well to a wide variety of OARs.

**Table 1:** A list of features used for each model.

Single-contour feature models (Z-score, MD, AE)					Connectedness model	CCR model
Location features	Orientation features {0<x<1}	Volume features	Shape features	CT number features	Missing slices or ‘ditzel’ features	Relational features
Centroid X [mm]	${\text{PC}1}_{\hat{x}}$	Volume [cc]	${\text{λ}}_{PC2}/{\text{λ}}_{PC1}$ *{0<x<1}*	CT minimum	Number of disconnected parts	Minimum distance [mm] *{0<x<∞}*
Centroid Y [mm]	${\text{PC}1}_{\hat{y}}$		${\text{λ}}_{PC3}/{\text{λ}}_{PC1}$ *{0<x<1}*	CT maximum		Fractional volume overlap *{0<x<1}*
Centroid Z [mm]	${\text{PC}1}_{\hat{z}}$		X extent (mm)	CT mean		
	${\text{PC}2}_{\hat{x}}$		Y extent (mm)	CT std. dev.		
	${\text{PC}2}_{\hat{y}}$		Z extent (mm)			
	${\text{PC}2}_{\hat{z}}$		Sphericity *{0<x<1}*			

The centroid features in the lateral, vertical, and longitudinal directions (defined as positive x, y, and z respectively) were calculated as the difference between the contour’s centroid and the brainstem’s centroid. For brainstem contours, it was calculated as the difference in centroid locations between the brainstem contour and the pituitary contour. This accounted for variations in image coordinates between CT images. The brainstem contour was chosen because of its central location and because it is anatomically present in every patient. The extent in x, y, and z was calculated as the difference between the largest and smallest pixel coordinate values for a given contour. Principal component analysis was performed to obtain the eigenvectors and eigenvalues of the principal components (PC) of a contour’s pixel coordinates. The x, y, and z components of the first and second PC eigenvectors were used as orientation features, while the ratio of the second and third PC eigenvalues (λ) to the first were used as shape features.

The orientation of a PC eigenvector can be arbitrarily positive or negative (for example $\vec{v}$ = +0.58 $\hat{x}$ +0.58 $\hat{y}$ +0.58 $\hat{z}$ or $\vec{v}$ = -0.58 $\hat{\;x}$ -0.58 $\hat{y}$ -0.58 $\hat{z}$ ). To standardize the orientation of PC1 or PC2 vectors for a given OAR, we identified a representative eigenvector ${\vec{r}}_{OAR}$ from the training set using Equation 1.


(1) $${\vec{r}\;}_{OAR}=\underset{\vec{v_{i}}\in V_{OAR}}{\text{argmax}}\left(\sum_{j\neq i}^{n}\left|{\vec{v}\;}_{i}\;\cdot {\vec{v}\;}_{j}\right|\right)$$

Where $V_{OAR}$ is the set of all PC1 or PC2 eigenvectors for a given OAR type in the training set with number n and $\vec{v}\;$ is a single PC eigenvector. After identification of ${\vec{r}\;}_{OAR}$ , the orientation of all eigenvectors in the training, validation, and test set ( $V_{OAR}^{\;*}$ ) were oriented either positive or negative to maximize the dot product between ${\vec{r}\;}_{OAR}$ and each eigenvector $\vec{v}\;\in V_{OAR}^{\;*}$ .

Since outlier detection approaches that use one-class training are sensitive to outliers in the training dataset, an outlier removal technique was applied to the training dataset after feature selection. To do this, the training dataset consisting of only acceptable contours was grouped based on its OAR type, and the median absolute deviation (MAD) from the median was calculated for single-contour features. A contour was excluded from the training set if any of its single-contour features deviated from the median by more than twelve MAD. This resulted in the removal of 0% to 3.6% of contours from the training set for each OAR type. The threshold of twelve MAD was determined by evaluating the number of contours removed for each OAR type and the impact of contour removal on model performance for the validation dataset.

### 2.5 Single-contour feature models

After single-contour feature calculation using acceptable contours in the training dataset, an individual model was trained for each OAR type for three single-contour feature model types (z-score, MD, and AE models). The z-score model calculated individual feature z-scores using Equation 2.


(2) $$z=\left|\frac{x-\mu }{\sigma }\right|$$

Where μ and σ are the mean and standard deviation of feature values in the training set. After calculation, the maximum z-score value across all the features is selected as the output metric. The MD model used the Mahalanobis distance of a contour’s features with respect to the training dataset features as the output metric (17, 18) and is calculated using Equation 3.


(3) $$D\left(\vec{x}\right)=\sqrt{{\left(\vec{x}-\hat{\mu }\right)}^{T}{\text{Σ}}^{-1}\left(\vec{x}-\hat{\mu }\right)}$$

Where $\hat{\mu }$ is a vector containing the mean feature values and ${\text{Σ}}^{-1}$ is the inverse of the covariance matrix calculated from the training data set. The output metric for the AE network was the mean squared difference between reconstructed features and input features for a given contour (19). The AE network was trained in MATLAB^®^ using the ‘trainAutoencoder’ function and consisted of a single hidden layer with 18 neurons and a cost function with a single L2 regularization term. To standardize the feature set, feature z-scores were calculated for input into the AE model. The number of epochs was limited to a maximum of 7000, and the L2 weight regularization coefficient was set to 0.005. The number of hidden layers and L2 regularization coefficient were optimized by evaluating model performance on the validation set across a range of values. The results of each single-contour feature model type were assessed individually and in combination with CCR and connectedness models for the validation and test set.

### 2.6 CCR model

For the CCR model, our objective was to come up with a set of features that could quantify varying degrees of contour-to-contour overlap and separation. To do this, the CCR model utilized the minimum distance between two contours and the fractional volume of overlap of one contour with another as its features. The combination of both features yielded all the information needed to quantify these relationships. A boolean matrix with 42 rows and 43 columns was generated to select the CCRs to include in the CCR model. Rows were associated with the selected contour, while columns were associated with the comparison contour. An additional column was added to allow comparison to the body contour (**Supplementary Figure S3**). The selected CCRs primarily focused on OARs that were close to each other. This included OAR types with distinct anatomical boundaries (e.g., cord and brain stem) and cases where one OAR was a subset of another (e.g., brain stem and brain). Well-defined contours in these cases should exhibit consistent anatomical boundaries with each other. In contrast, contours that are not in close proximity to each other may have more uncertainty in their relationship, making them susceptible to false positives.

The minimum distance feature data was fit to a gamma distribution (22) ranging from zero to infinity, while the fractional overlap volume feature data was fit to a beta distribution (23) ranging from zero to one. Distribution types were selected to have the same upper and lower input domains as their representative features and followed the probability distribution of the CCR features. Initial upper and lower outlier cutoffs were determined by taking the upper and lower 99^th^ percentile boundaries of the fitted distribution. The percentile boundaries were set manually to minimize the number of false positives detected by the CCR model in the validation set. The determined percentile boundary cutoffs were expanded by 0.02 for fractional volume and 2mm for minimum distance to minimize identification of errors that were present, but small enough to not be clinically relevant.

### 2.7 Connectedness model

For human reviewers, identifying disconnected voxels in a contour can be time-consuming. To improve clarity for potential human reviewers using this QA tool, we separated the connectedness feature from the single-contour feature models and created a separate model including only the number of connected parts in a contour. This enables easy reporting of this feature to reviewers. To establish the maximum number of allowable parts, a statistical threshold of 99.95% was set using a gamma distribution fitted to the training data. The threshold was optimized by evaluating performance on the validation dataset and selected to minimize false positives. A statistical threshold was used instead of setting a predetermined cutoff as some contours were allowed to have multiple parts anatomically (e.g. thyroid) and other contours could have multiple parts due to CT image-related scan truncation (e.g. left and right brachial plexus).

### 2.8 Model combination

To obtain the final combined classifications, if any individual model identified a contour as erroneous, it was classified as such. Thresholds for the connectedness model and CCR model output metrics were set manually and the single-contour features model thresholds were tuned to maximize balanced accuracy for the combined classifications (24). Balanced accuracy is defined as the average of sensitivity and specificity. While accurate detection of erroneous contours is more clinically relevant, the prevalence of erroneous contours will typically be low in the clinical workflow. We estimate a reasonable prevalence of erroneous contours in the clinical workflow to be 10% and the relative severity of incorrectly categorizing erroneous compared to acceptable contours at 9 to 1. In this case, balanced accuracy will be an appropriate optimization metric (25). The values of prevalence and relative severity can easily be adjusted, resulting in different optimal thresholds for future clinical use. The performance of the single-contour feature models without combination with CCR and connectedness models was also evaluated using the same threshold tuning. Thresholds for individual and combined single-contour feature models were not necessarily the same. The test set was assessed using the same thresholds obtained from the validation set.

### 2.9 Statistics

To reduce class imbalance during statistical assessment, we adopted a solution involving random subsampling. Specifically, we selected five acceptable contours at random from the input curated gold-standard contours for each OAR type and merged them with the erroneous validation contours. This approach allowed us to present a single statistical test that was more evenly balanced in terms of its evaluation of performance on both acceptable and erroneous contours. The subsampling included 210 acceptable and 190 erroneous contours. Statistical testing of model performance was performed using the two-sided mid-p value McNemar test with a p-value of less than 0.05 considered to be significantly different (26, 27).

## 3 Results

### 3.1 Single-contour feature method comparison and model combination

**Figure 2:**
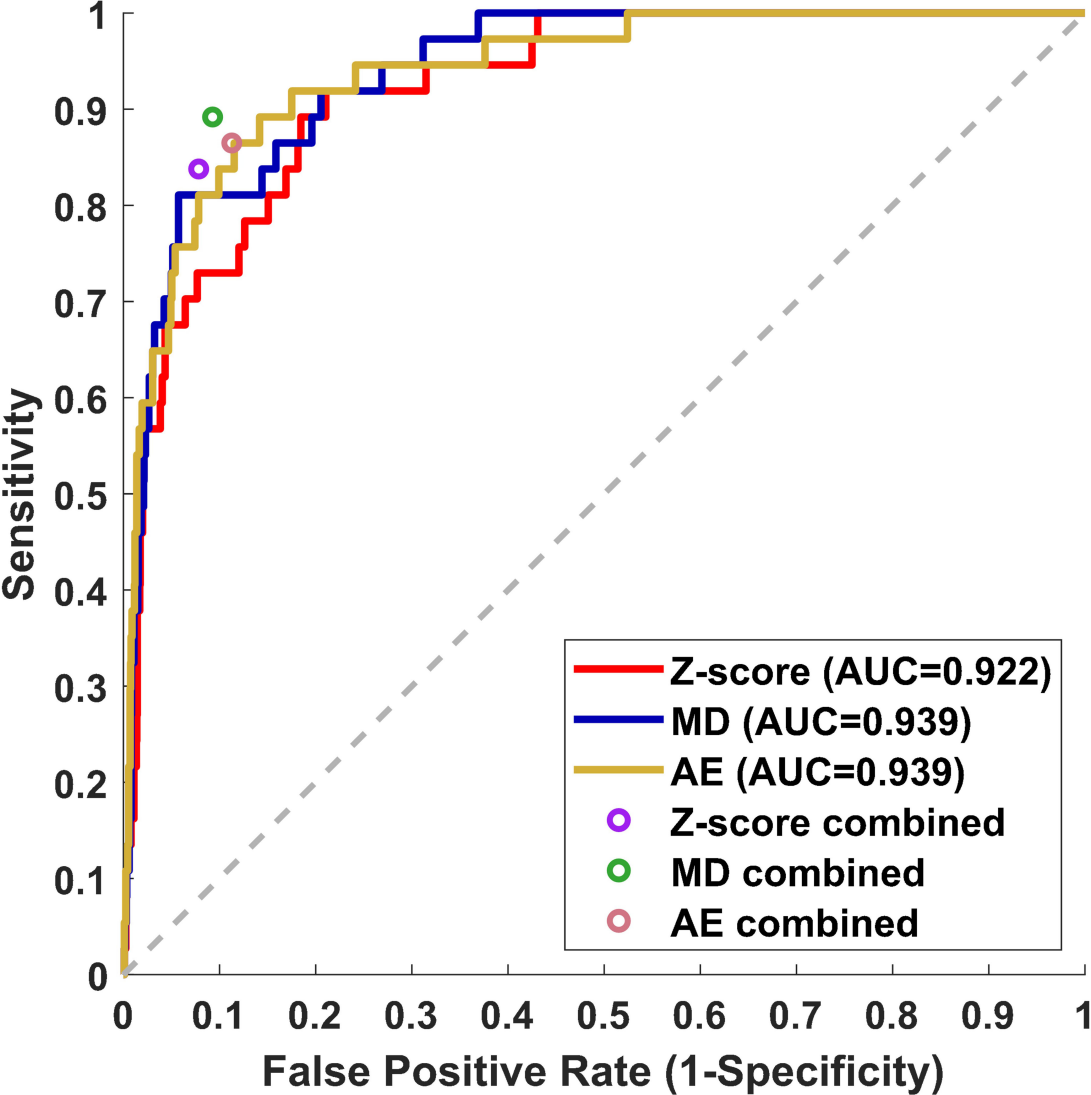
Receiver operating curve (ROC) results from the test dataset for three individual single-contour features models, z-score, MD, and AE models. The false positive rate and sensitivity of single-contour feature models combined with CCR and connectedness models are plotted as circles on the ROC plot.

Receiver operating curves were used to evaluate the performance of individual z-score, MD, and AE models for all contours. The z-score, MD, and AE models had an area under the curve (AUC) values of 0.922, 0.939, and 0.939 respectively for the test set (**Figure 2**, **Table 2**). Combining the single-contour feature models with CCR and connectedness models led to improved performance for all three single-contour feature models. The high specificity of the CCR (0.982) and connectedness (0.990) models made it possible to combine them by identifying a contour as an outlier if any of the models flagged it as one (logical OR) with minimal decrease in combined model specificity. Test set results were similar to the validation set for all models, indicating minimal overfitting due to the single feature selection, model thresholding, and hyperparameter tuning using the validation dataset. In the statistical subset of the validation data, combination of the CCR and connectedness models with the single-contour feature models significantly improved the performance of the z-score (P=0.0007), MD (P=0.0175), and AE (P=0.0201) models (**Supplementary Table S2**), demonstrating the added benefit of incorporating CCR features for outlier detection.

**Table 2:** Classification results.

Model	AUC	Balanced Accuracy	Sensitivity	Specificity	True positive	False negative	True negative	False positive
Validation Set								
Connectedness	–	0.527	0.063	0.991	12	178	1966	18
CCR	–	0.730	0.474	0.987	90	100	1958	26
Z-score	0.852	0.794	0.684	0.904	130	60	1793	191
MD	0.899	0.826	0.811	0.842	154	36	1671	313
AE	0.896	0.838	0.763	0.913	145	45	1812	172
Z-score combined	–	0.866	0.816	0.916	155	35	1818	166
MD combined	–	0.882	0.842	0.921	160	30	1828	156
AE combined	–	0.884	0.863	0.906	164	26	1797	187
Test Set								
Connectedness	–	0.522	0.054	0.990	2	35	1984	21
CCR	–	0.721	0.459	0.982	17	20	1969	36
Z-score	0.922	0.816	0.730	0.903	27	10	1811	194
MD	0.939	0.851	0.865	0.837	32	5	1679	326
AE	0.939	0.866	0.838	0.893	31	6	1791	214
Z-score combined	–	0.880	0.838	0.922	31	6	1848	157
MD combined	–	0.900	0.892	0.907	33	4	1819	186
AE combined	–	0.876	0.865	0.887	32	5	1779	226

### 3.2 Model performance across OAR types, error types, and error severity

**Figure 3:**
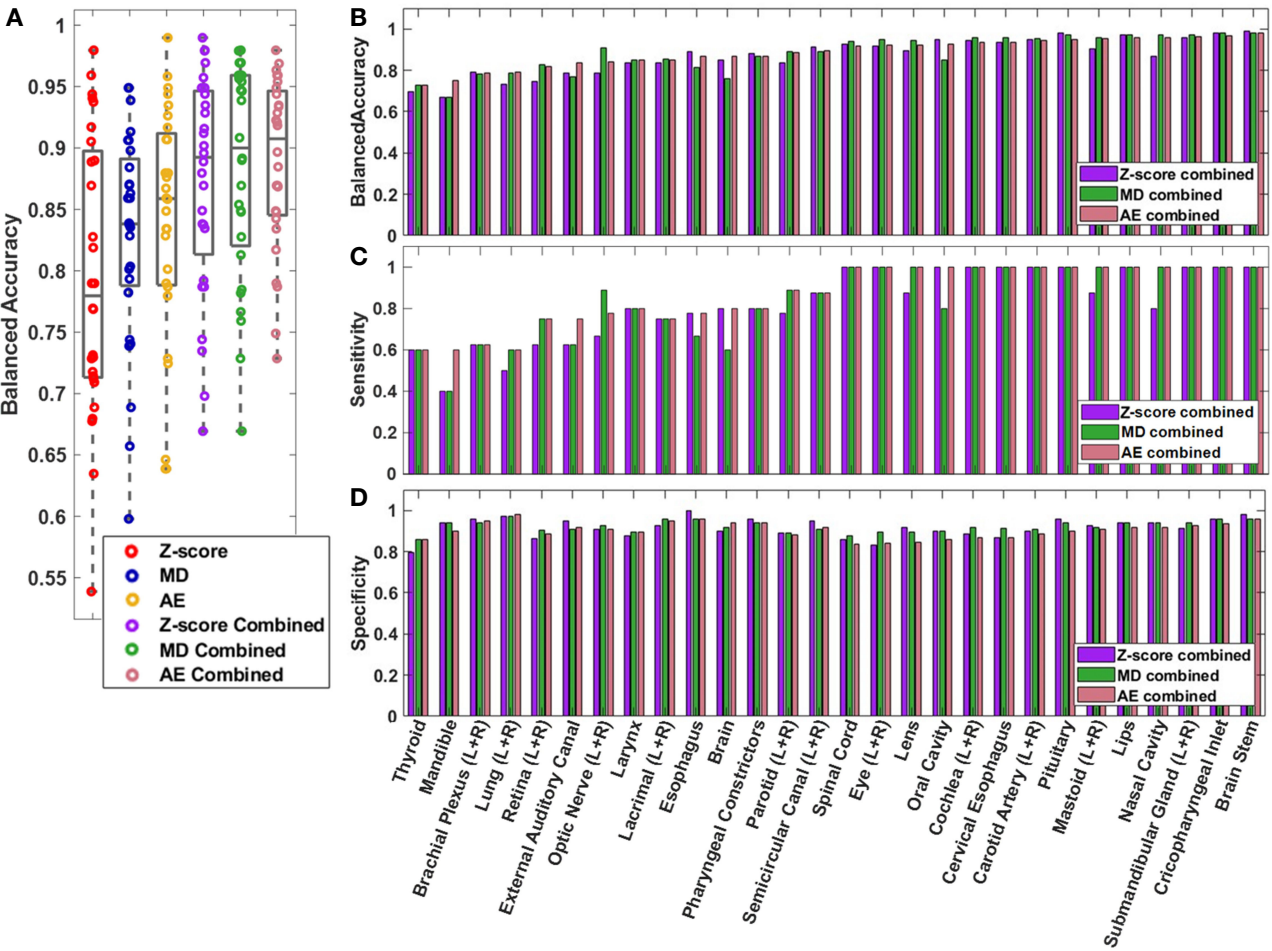
**(A)** Box and whisker plot (Box-inner quartile range, whisker-range) of balanced accuracy for each OAR type in the validation dataset. Left and right matching OARs were combined before plotting. **(B)** Balanced accuracy, **(C)** sensitivity, and **(D)** specificity are plotted for each OAR for the combined models.

**Figure 4:**
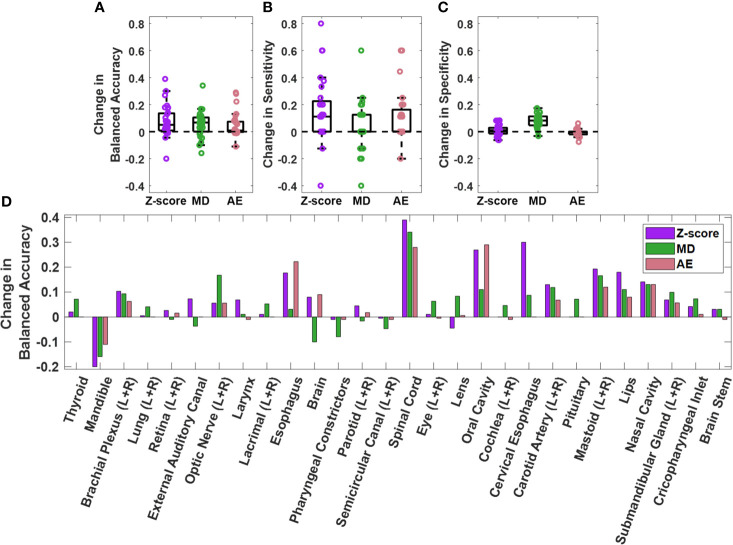
Change in **(A)** balanced accuracy, **(B)** sensitivity, and **(C)** specificity for single contour feature models on the validation dataset when single contour feature models are combined with CCR and connectedness models. Each datapoint on the plots represent an OAR type. Change for each OAR type individually is also plotted **(D)**. Positive change indicates improvement in performance after model combination.

The performance of the knowledge-based QA framework for each OAR type individually was evaluated using the validation dataset without distinguishing between left and right-sidedness for bilateral OARs (**Figure 3**, **Supplementary Figures S4**, **S5**). Analysis of the validation set allowed for an adequate number of contours of each OAR type to be available for classification evaluation. Of the three combined single-contour feature models, the combined AE model had both the highest median and highest minimum BA across all OAR types (Minimum median and maximum BA of 0.729, 0.908, and 0.980 respectively). Combining the single-contour feature models with CCR and connectedness models resulted in an average increase of 0.077 (z-score), 0.055 (MD), and 0.048 (AE) in BA values per OAR type. The improvements in BA were not evenly distributed across all OAR types. The AE model type showed the largest improvements for the spinal cord and oral cavity, while the mandible experienced worse performance (**Figure 4**), attributable to changes in the optimal single-contour feature thresholds when combined with CCR and connectedness models.

**Figure 5:**
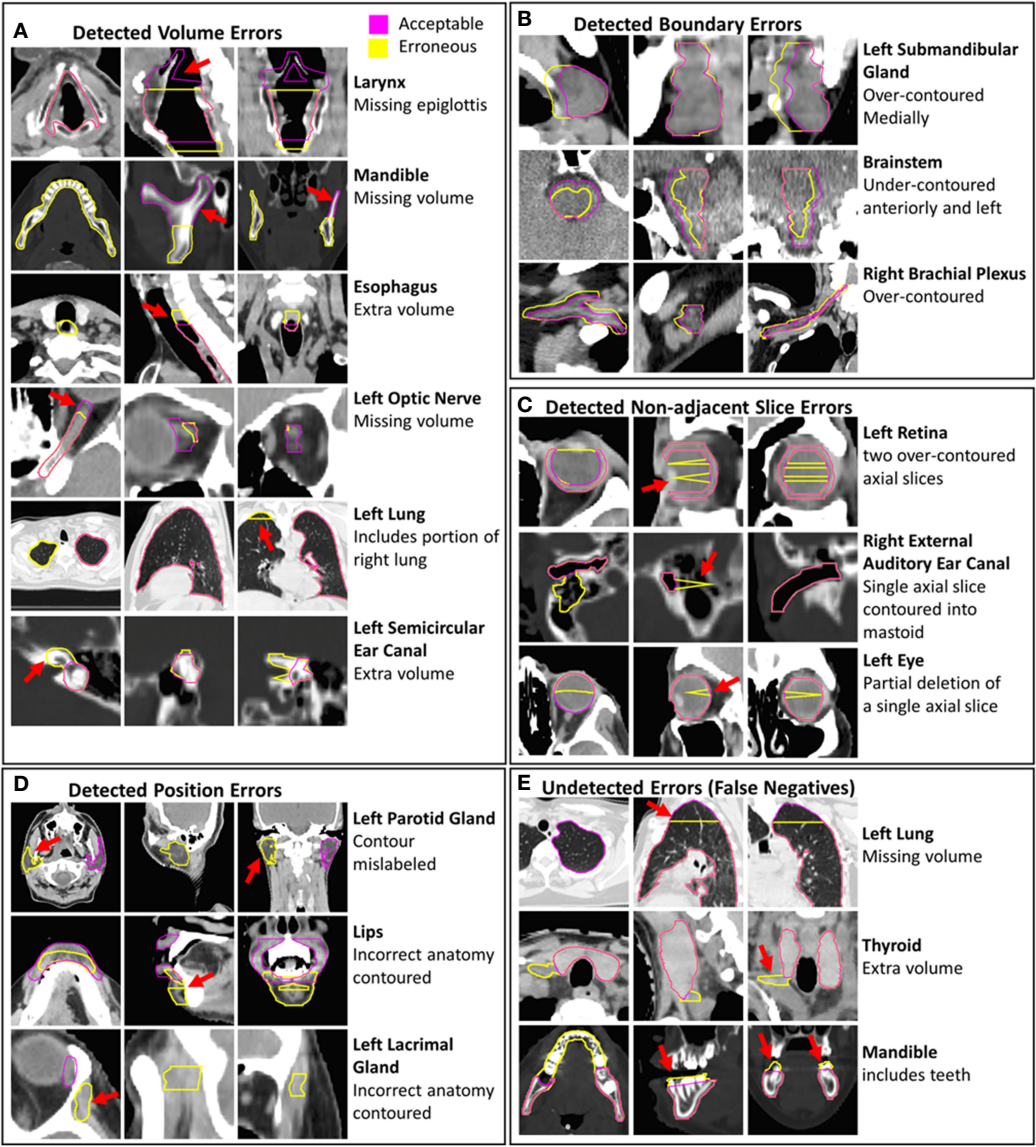
Examples of erroneous contours with volume, boundary, non-adjacent slice, and position errors are shown **(A–D)**. Examples of undetected contour errors (false positives are shown **(E)**. The corresponding gold-standard acceptable contours that were edited to create erroneous contours are displayed. Hounsfield unit display ranges were -10 to 70 for images with brainstem contours, -250 to 1500 for images with contours of bone, -1000 to 100 for images with lung contours and -115 to 115 for all other images.

The sensitivity of the combined AE model for boundary, position, non-adjacent slice, and volume error types was 0.867, 0.971, 0.833, and 0.8116 in the validation set. Similar classification accuracies across different error types were observed between combined AE, MD, and z-score models in both the test and validation sets (**Supplementary Table S3**). Higher sensitivity was observed for position errors compared to the other types of errors across all three combined model types, likely due to position errors tending to be more severe than other error types (**Figures 5A–D**).

The sensitivity of the combined AE model for detecting major and moderate errors was 0.922 and 0.810. Similar differences in sensitivity between major and moderate errors were observed for the combined MD and z-score models (**Supplementary Table S4**), suggesting that more sever errors are more likely to be detected by the knowledge-based QA framework.

### 3.3 Misclassifications

Some erroneous esophagus, lung, and brachial plexus contours that were incorrectly classified by all models had missing volumes (**Figure 5E**). These OARs are commonly affected by CT scan truncation which increases variation in volume and shape features. Additional undetected errors included improper boundary delineation (either over-contouring or under-contouring boundary edges) and volume changes that were small relative to the total volume of the contour. Some acceptable contours were identified as erroneous by all combined models. These included contours with clinically insignificant inaccurately delineated boundaries, contours that were anatomically accurate but contoured on patients with abnormal positioning or anatomy, and contours on CT scans with metal artifact-related image quality issues (**Supplementary Figure S6**). The CCR model was able to identify outliers from improper separation or overlap (**Supplementary Figure S7**). Out of the 34 erroneous contours from the validation set that were missed by all individual single-contour feature models, the CCR model identified 15.

## 4 Discussion

We have developed a knowledge-based method for detecting clinically relevant erroneous OAR contours in HN radiotherapy. Our method uses models based on single-contour features, as well as CCRs. Combining the single-contour feature model with the CCR and connectedness models significantly improves performance for the z-score, MD, and AE models. The combined AE model achieves a sensitivity of 0.865, specificity of 0.887, and BA of 0.876 for the test set. Similar BA, sensitivity, and specificity were observed for the combined z-score, MD, and AE models for both test and validation datasets, indicating no overfitting in the validation set. Minimum, median, and maximum balanced accuracies across individual OAR types for the AE model were 0.729, 0.908, and 0.980, respectively on the validation set. Our results demonstrate satisfactory model performance for a comprehensive set of OAR types utilized in HN radiotherapy.

Accurately detecting contour errors across a wide range of OAR types is a significant challenge. Many studies examining model performance have been limited to assessing no more than 17 OAR types (9, 10, 12, 14, 16). One study looking at pelvis, abdomen, and thorax regions reported results for 40 OAR types, however, their primary aim was to develop a method for classification of contours to an OAR type or label rather than to detect erroneous contours (15). Furthermore, they did not report the model performance for each OAR type, instead only reporting the overall AUC results. In our study, we use a knowledge-based outlier detection approach with a combined AE model that achieves a minimum sensitivity and specificity of 0.600/0.837 (ignoring left-right distinction) per OAR type for 42 HN OAR types used clinically. The wide variety of contour volumes, and shapes, as well as a large dataset of patients with several different HN disease types and sites, demonstrates that knowledge-based OAR QA for HN radiotherapy is both feasible and generalizable to a wide variety of OARs.

Abnormalities in CT images, caused by factors like CT artifacts, patient positioning, or abnormal anatomy, can contribute to higher false positive rates for knowledge-based outlier detection. However, these images may still result in suboptimal quality for both human-generated and DL-generated contours, highlighting the importance of careful manual review in such situations. Although the knowledge-based quality assurance system may yield false positives when encountering abnormal image data with accurate contours, it provides a rapid and efficient method to aid reviewers in automatically identifying erroneous contours.

The CCR model is a novel tool that can identify incorrect amounts of overlap or separation between two contours. This is crucial in clinical settings for two reasons: first, overlap and separation should be consistent with actual anatomy, and second, gaps between contours that are anatomically touching may result in unreported high doses to the OAR. In this study, we chose CCRs that had consistent anatomical relationships or were close to each other, but the technique can be extended to any CCRs. The high specificity of the CCR model allows for easy deployment as a contour review tool, either on its own or in conjunction with other models.

One limitation of this study is that CCR calculations for both erroneous and acceptable contours were performed only in relation to acceptable contours and never in relation to erroneous contours. This facilitated the identification and quantification of the CCRs model performance. In a real-world application, the CCR model will only detect incorrect CCRs instead of directly identifying incorrect contours. Therefore, in clinical practice, the end user would need to review two contours for each improper CCR to identify a single unacceptable contour.

The exclusion of data from the training set based on the number of MAD from the median provides a way to remove contours of questionable quality in the training dataset. The threshold for data removal can be tuned with a validation dataset. In this work, increases in balanced accuracy for the combined models when implementing outlier removal ranged from 0.00 to 0.06 depending on the model used. For less curated datasets, this approach may have a larger impact on model performance and help improve the generalizability of the QA framework to different datasets.

The knowledge-based QA framework presented in this work has the potential to improve the detection of erroneous contours when used in conjunction with human reviewers. This will require an efficient integration within the clinical contour review workflow, where the QA framework results can be quickly accessed and interpreted by a human reviewer. A script-based approach run directly from the clinical contouring software would be an effective option. This script could allow human reviewers to automatically archive human review labels, model inferences, and contours when run. This data archiving would facilitate model performance tracking, iterative model improvement, and the assessment of the dosimetric impact of erroneous contours.

The use of a large, highly curated HN OAR dataset for model development is a clear foundational strength of our study. However, our modeling also required erroneous H&N contours. This data was not available *a priori*, necessitating fabrication; we recognize that this could be perceived as a weakness in terms of presented model performance evaluation. Our immediate goal is to iteratively develop a clinical solution based on the presented methodology for integration within our contour review workflow. As we detect true erroneous contours during preliminary deployment phases, these erroneous OARs detected “in the wild” can be leveraged for future refinements (iterations in model training/tuning). Thus, we emphasize that the presented model framework, model comparison, and the generalizability of this approach to many OAR types should be recognized as the main focus of this study.

The best-performing combined AE. model can identify erroneous contours but does not identify individual features that are abnormal. To reduce the time spent during human review of contours marked as erroneous by the QA framework, it may be beneficial to identify specific abnormal features along with erroneous contours to guide reviewers more quickly to the errors in the contours. To obtain predictions on abnormal features after identifying erroneous contours using the combined AE model, a separate z-score model could be used *post hoc* to report outlier features. However, this approach may result in both models disagreeing on a contour’s classification. Alternatively, more sophisticated model-agnostic tests can be employed to determine the importance of input features in making predictions, which can be useful in identifying features that strongly influence model decisions (28, 29). Additional research is needed to determine whether the identification of erroneous features in this manner would reduce contour review time.

Further research is necessary to evaluate the developed QA framework for other anatomical sites. The QA framework can be extended to other treatment regions with additional sets of curated and outlier data given its adaptability to a variety of OARs for HN. However, it is anticipated that the performance of the CCR model may decrease in the thorax, abdomen, and pelvis due to fewer consistent anatomical relationships between OARs. A better understanding of the amount of curated data needed will become more apparent after the integration of the HN model into our clinical workflow.

In the future, model generalizability to other institutions also needs to be assessed. Several challenges are associated with this, including variation in contour definitions (7), and variation in the determination of clinically relevant contour errors between different institutions. While trained models could be directly deployed in outside institutions, outside institutions could also train their own institution specific model using the same QA framework as illustrated here. This would allow any differences in contour definitions, and contour error definitions to be accounted for. More research is needed to assess the generalizability of this approach to other institutions.

## 5 Conclusion

In this study, we have created a method for knowledge-based QA that utilizes single-contour features and contour-to-contour relationships to identify erroneous contours for forty-two HN OAR types. The effectiveness of multiple models has been evaluated, both in general and for each OAR type. The findings of this study demonstrate the developed framework for knowledge-based QA of HN contours is both feasible and generalizable to a full set of clinical HN OARs.

## Data availability statement

The datasets presented in this article are not readily available because of Mayo Clinic policies on data use and sharing. Requests to access the datasets should be directed to brooks.jamison@mayo.edu.

## Ethics statement

The studies involving humans were approved by Mayo Clinic Rochester Minnesota USA. The studies were conducted in accordance with the local legislation and institutional requirements. Written informed consent for participation was not required from the participants or the participants’ legal guardians/next of kin in accordance with the national legislation and institutional requirements.

## Author contributions

JB: Conceptualization, Data curation, Formal analysis, Investigation, Methodology, Software, Validation, Visualization, Writing – original draft. ET: Conceptualization, Data curation, Investigation, Methodology, Supervision, Writing – review & editing. AA: Data curation, Writing – review & editing. CB: Data curation, Writing – review & editing. RF: Conceptualization, Data curation, Writing – review & editing. JL: Conceptualization, Data curation, Methodology, Software, Writing – review & editing. NL: Data curation, Writing – review & editing. DR: Conceptualization, Writing – review & editing. SP: Data curation, Writing – review & editing. SS: Data curation, Software, Writing – review & editing. DM: Conceptualization, Data curation, Formal analysis, Investigation, Methodology, Software, Supervision, Writing – review & editing.
