# Supplementary material for: Knowledge-based quality assurance of a comprehensive set of organ at risk contours for head and neck radiotherapy
Source: Front Oncol. 2024 Feb 29;14:1295251. doi: 10.3389/fonc.2024.1295251 (PMC10937434; doi:10.3389/fonc.2024.1295251)
Supplement: Supplementary file 1 [file DataSheet_1.docx]

Supplementary Material

Knowledge-based quality assurance of a comprehensive set of organ at risk contours for head and neck radiotherapy

Jamison Brooks^1^, Erik Tryggestad^1^, Aman Anand^2^, Chris Beltran^3^ Robert Foote^1^, J. John Lucido^1^, Nadia N. Laack^1^, David Routman^1^, Samir H. Patel^2^, Srinivas Seetamsetty^1^, Douglas Moseley*^1^

^1^Department of Radiation Oncology, Mayo Clinic Rochester, Rochester, MN, USA

^2^Department of Radiation Oncology, Mayo Clinic Arizona, Phoenix, AZ, USA

^3^Department of Radiation Oncology, Mayo Clinic Florida, Jacksonville, FL, USA

*** Correspondence:**

Douglas Moseley*

[*Moseley.Douglas@mayo.edu*](mailto:Moseley.Douglas@mayo.edu)

# Supplemental Methods

The majority of CT scans were performed with several Somatom Definition AS CT scanners that are tested monthly for HU constancy to ensure consistent HU values. Initial measurements are performed at commissioning to baseline the characteristics of the CT scanners and verify they are within vendor specifications for HU linearity. Monthly testing is then performed using the CTP404 module in a CatPhan® phantom to ensure HU value constancy relative to the time of commissioning. This is done with material inserts including acrylic, air, polystyrene, low density polyethylene, polymethylpentene, teflon, and delrin (Supplemental Table 1).

# Supplemental Tables

**Supplemental Table S1:**  The range of acceptable HU value variation from baseline allowed during monthly quality assurance testing for one of the CT scanners used in this work.

| Material | Nominal value | Action level |
| --- | --- | --- |
| Acrylic | 115 | ±6 |
| Air | -1000 | ±50 |
| Polystyrene | -47 | ±6 |
| LDPE | -104 | ±5 |
| PMP | -196 | ±8 |
| Teflon | 1000 | ±50 |
| Delrin | 365 | ±16 |

Supplemental Table S2: Performance on subset of validation set used for Mcnemar’s test.

| Model | AUC | Balanced accuracy | Sensitivity | Specificity | True positive | False negative | True negative | False positive |
| --- | --- | --- | --- | --- | --- | --- | --- | --- |
| Validation set | | | | | | | | |
| Connectedness | - | 0.527 | 0.063 | 0.990 | 12 | 178 | 208 | 2 |
| CCR | - | 0.725 | 0.474 | 0.976 | 90 | 100 | 205 | 5 |
| Z-score | 0.857 | 0.802 | 0.684 | 0.919 | 130 | 60 | 193 | 17 |
| MD | 0.900 | 0.832 | 0.811 | 0.852 | 154 | 36 | 179 | 31 |
| AE | 0.896 | 0.841 | 0.763 | 0.919 | 145 | 45 | 193 | 17 |
| Z-score combined | - | 0.863 | 0.816 | 0.910 | 155 | 35 | 191 | 19 |
| MD combined | - | 0.874 | 0.842 | 0.905 | 160 | 30 | 190 | 20 |
| AE combined | - | 0.877 | 0.863 | 0.890 | 164 | 26 | 187 | 23 |

Supplemental Table S3: Classification performance for combined models for each type of contouring error.


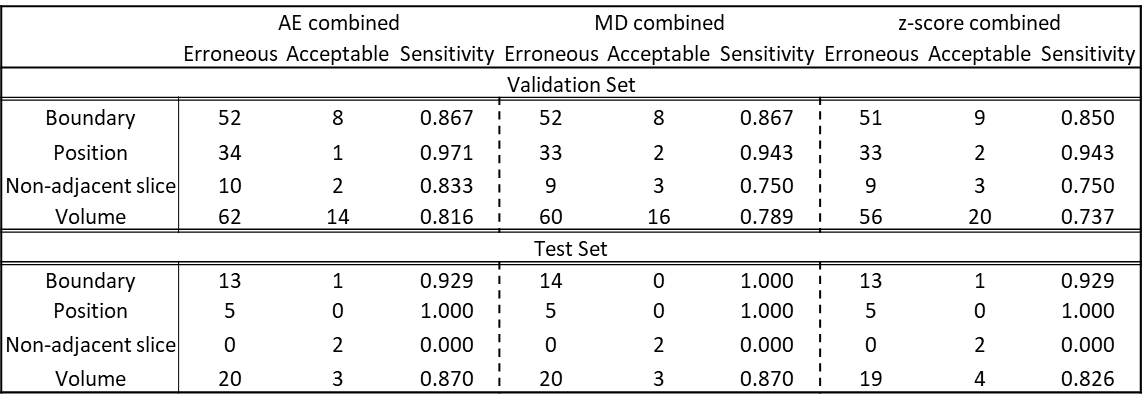


**Supplemental Table S4:** Classification performance for combined models according to Error Severity


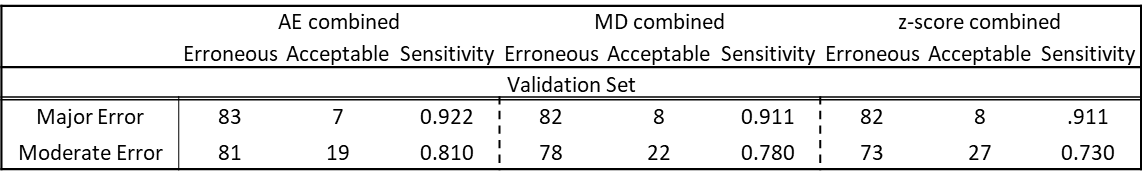


# Supplemental Figures

#
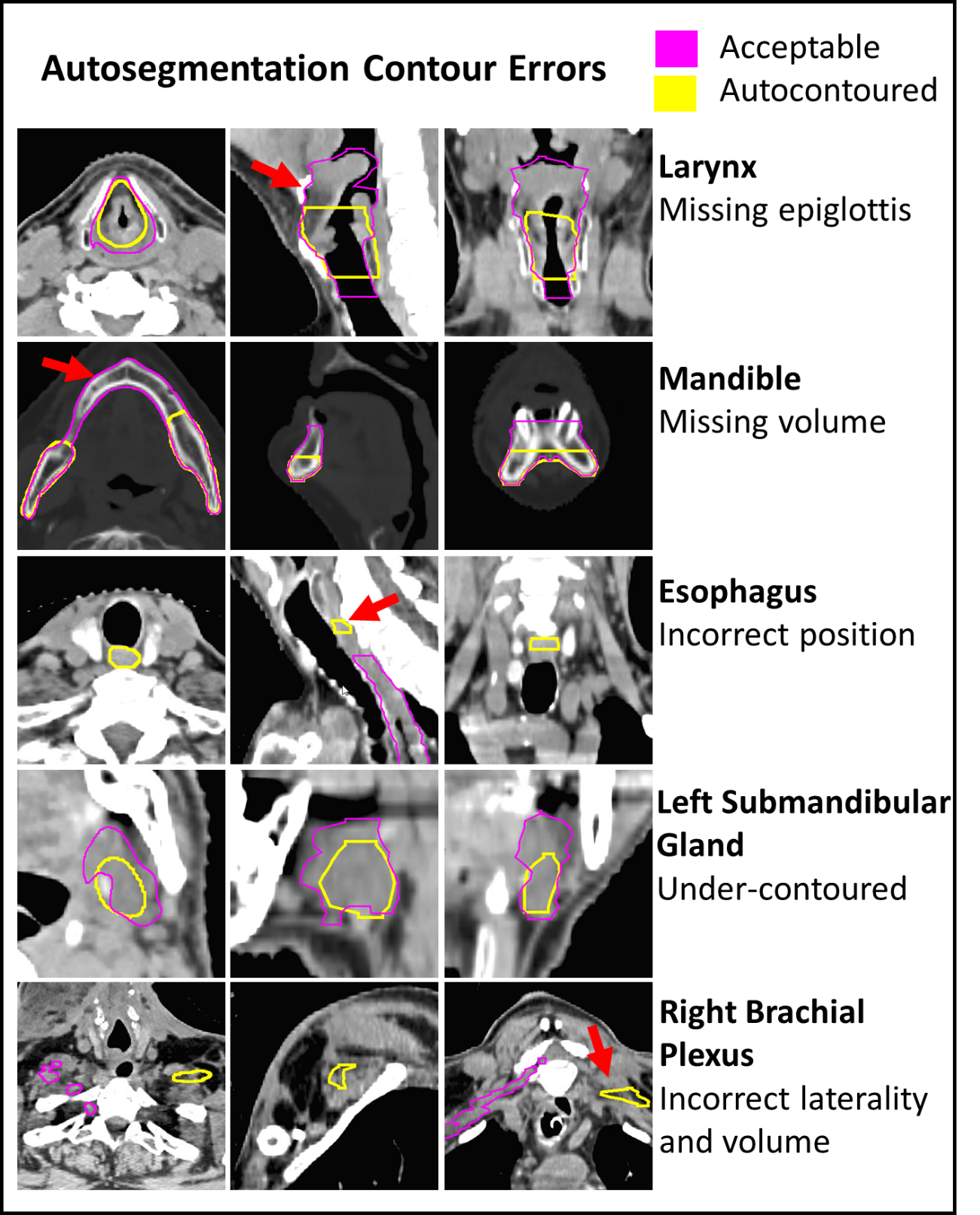


# Supplemental Figure S1: Examples of HN contours from five commercially available autocontouring software packages approved for clinical use by the FDA and acceptable manually created gold-standard contours are shown in yellow and magenta, respectively. Hounsfield unit display ranges were -115 to 115 for images of soft tissue contours and -250 to 1750 for bone images.


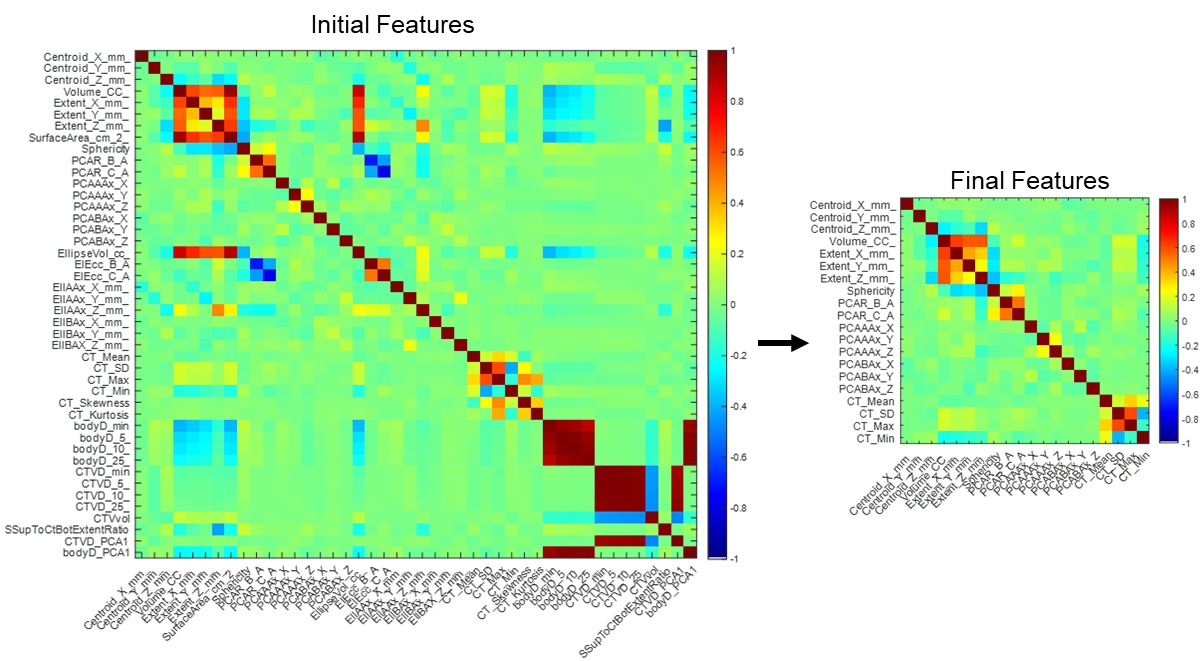


Supplemental Figure S2: Pearson correlation coefficient matrix for the 44 features selected initially (left) and final selected features (right). Average feature correlation across all OAR types is shown. CTVD_min, CTVD_10, CTVD_25, CTVD_PCA1, bodyD_min, bodyD_5, bodyD_10, bodyD_25, bodyD_PCA1 features represent overlap volume histogram-based features of a contour with the CTV and body contours. EllaAx_X_mm, EllAAx_Y_mm, EllAAx_Z_mm, EllBAx_X_mm, EllBAx_Y_mm, EllBAx_Z_mm represent the lengths in x, y, and z directions for the first and second principal axis of the minimum volume ellipsoid that encompasses a given contour. EllipseVol_cc is the volume of the minimum volume bounding ellipse. EllEcc_B_A and EllEcc_C_A are ratios of the magnitude of the principal axis of the minimum volume of the bounding ellipse. SSupToCtBotExtentRatio is the ratio of the extent of a contour in the z direction to the distance in the z direction from the superior side of the contour to the bottom of the CT image.

Supplemental Figure S3: CCR matrix. Rows correspond to the contour that is evaluated, columns correspond to comparison contours. CCRs to check are indicated in orange and marked with a 1. CCRs were selected by physician and physicist experience and institutional guidelines. The number of checks per OAR are displayed in parenthesis after the evaluated OAR name.


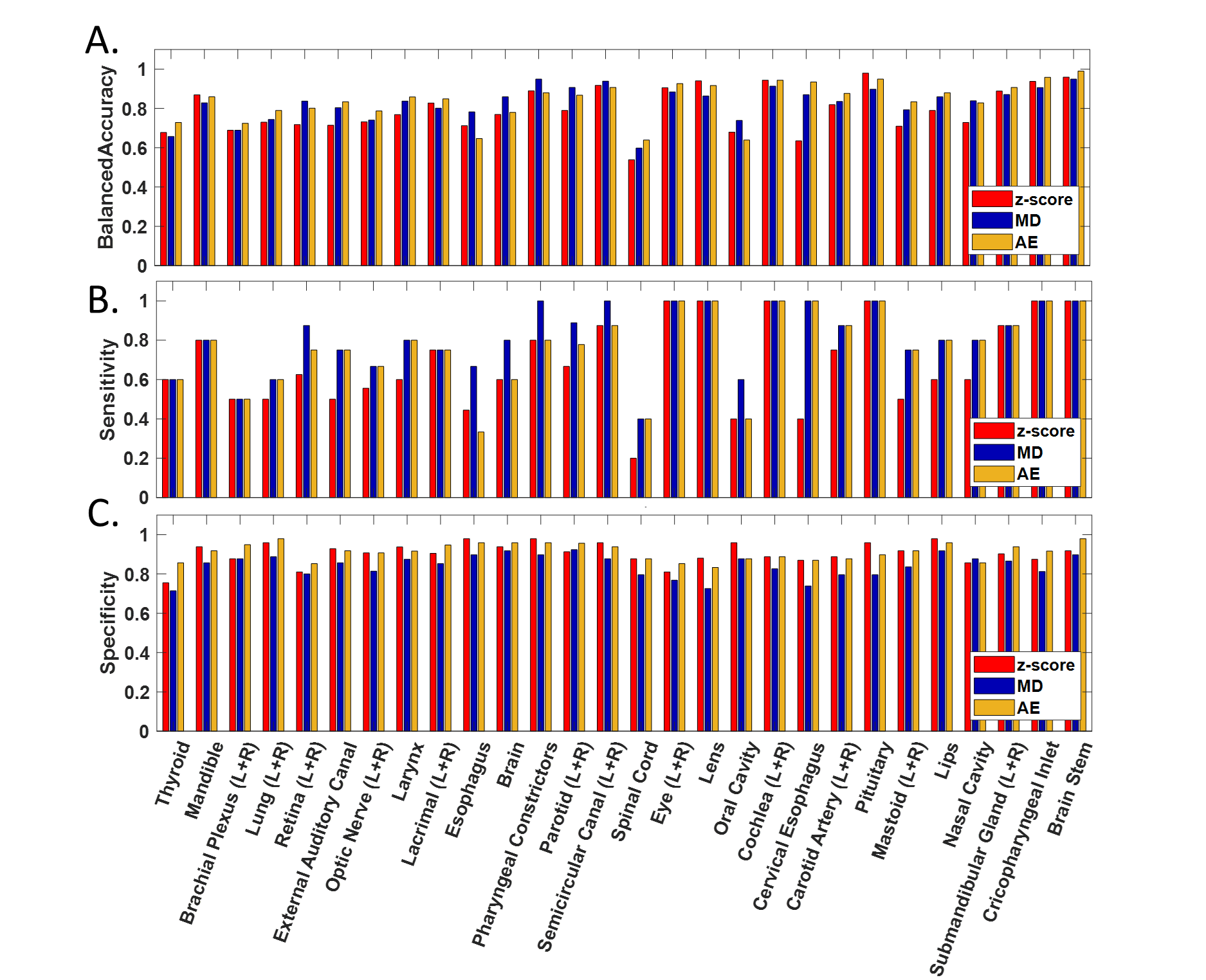
Supplemental Figure S4: Performance metrics for individual single contour feature models by OAR.


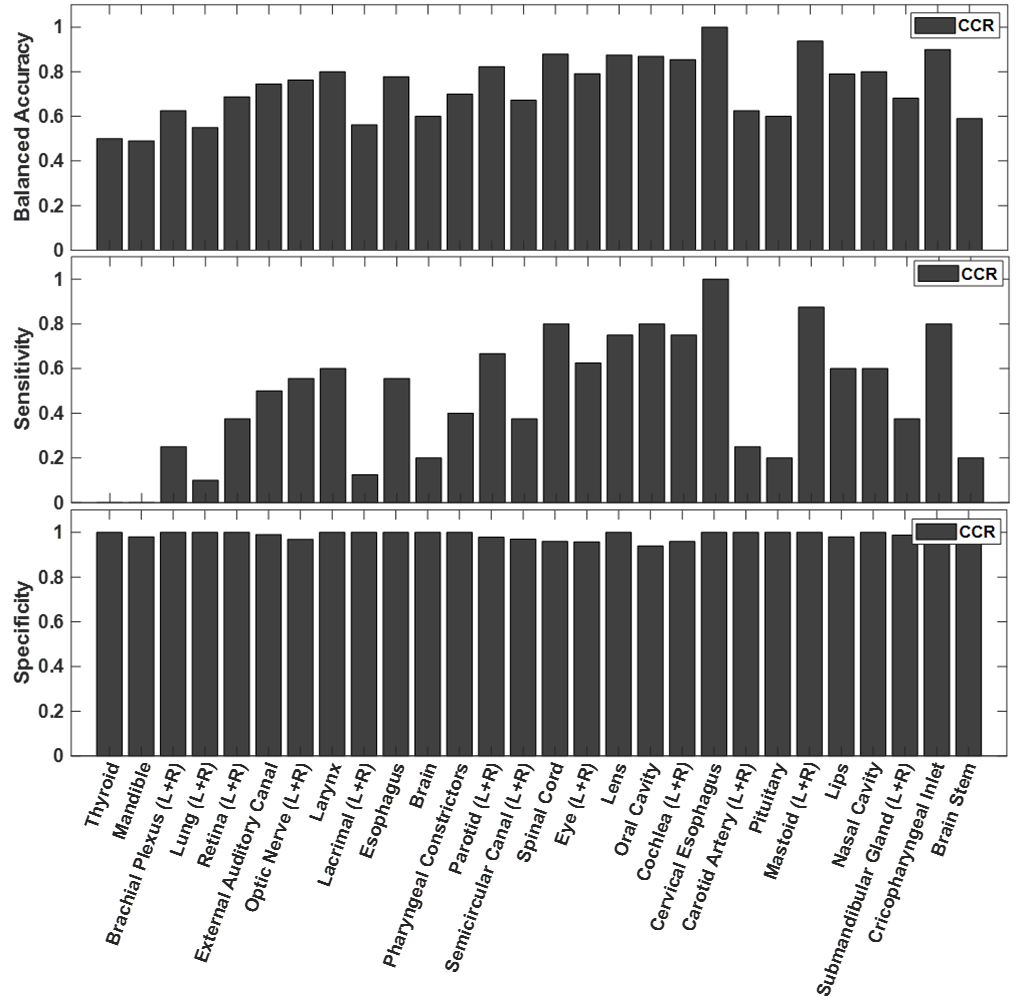


**Supplemental Figure S5:** Performance metrics for CCR model alone by OAR type.


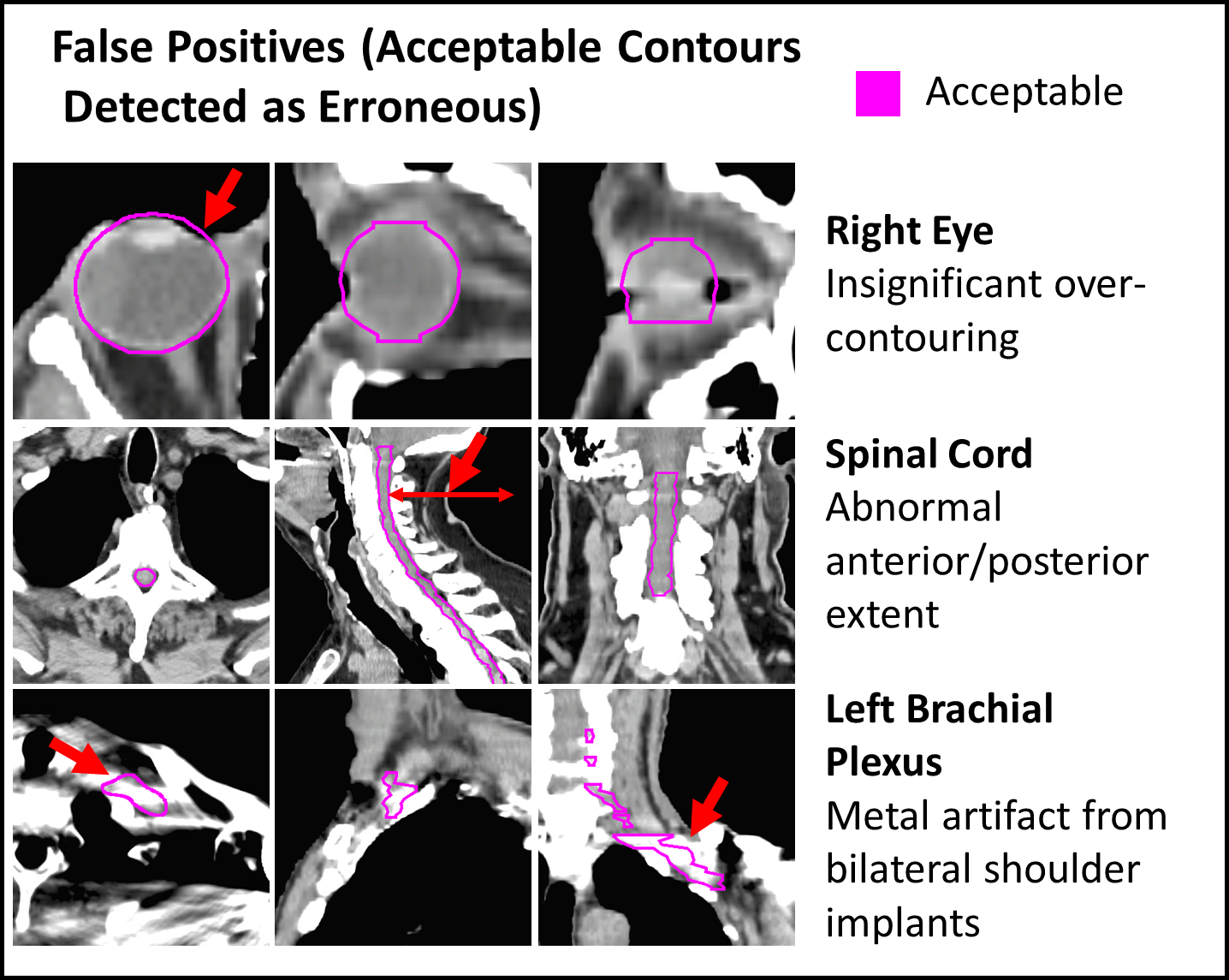


Supplemental Figure S6: Examples of acceptable gold standard contours that were flagged as erroneous by the AE combined model. Acceptable contours are shown in magenta. Hounsfield unit display ranges were -115 to 115 for all images.


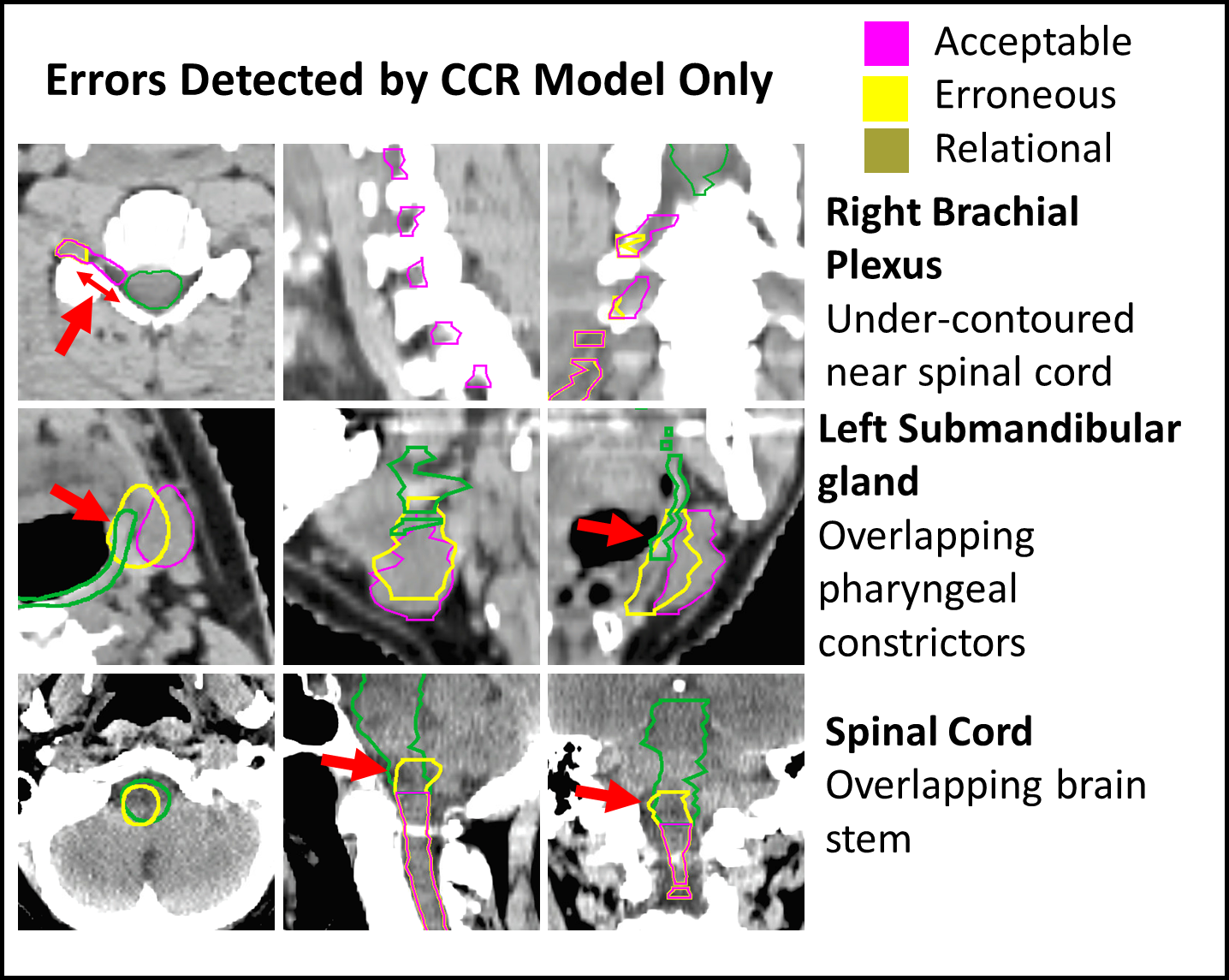


Supplemental Figure S7: Examples of errors that were detected by the CCR model that were not detected by individual AE, MD, or z-score models. Original acceptable contours are shown in magenta, erroneous contours derived from the original contours are displayed in yellow. Relational contours with abnormal overlap or separation are displayed in green. Hounsfield unit display ranges were -115 to 115 for images containing brachial plexus and submandibular gland and -10 to 70 images containing spinal cord contours.
